# Triple Valve Endocarditis With Aortic Root Abscess Presenting With Complete Heart Block and Distal Embolization

**DOI:** 10.7759/cureus.13942

**Published:** 2021-03-17

**Authors:** Mitra Patel, Zeid Nesheiwat, Neha Patel, Ronak G Soni, Mohammed Maaieh

**Affiliations:** 1 Internal Medicine, University of Toledo College of Medicine, Toledo, USA; 2 Internal Medicine, Promedica Health System, Toledo, USA; 3 Cardiovascular Medicine, University of Toledo College of Medicine, Toledo, USA; 4 Cardiology, Promedica Health System, Toledo, USA

**Keywords:** endocarditis

## Abstract

Infective endocarditis (IE) carries a high mortality rate. Consequently, the prognosis is poorer in patients with multiple valve involvement. Due to poor prognosis of patients with endocarditis, early diagnosis and management of these patients can be challenging in the clinical setting. We describe a case of a 45-year-old man who came in with bacteremia secondary to a diabetic foot ulcer. Electrocardiogram (EKG) showed complete third-degree heart block which rose suspicion for possible valvular abscess formation. Transthoracic echocardiogram (TTE) was performed and revealed vegetations on the aortic and mitral valve. A follow-up transesophageal echocardiogram (TEE) showed an abscess on the aortic valve along with vegetations on the mitral and tricuspid valve, the latter which was missed on TTE. The prompt utilization of TEE in detecting early and late mechanical complications of endocarditis is imperative in facilitating rapid clinical decision-making and early intervention. Patients with multi-valve endocarditis are at extremely high risk of complications and should be evaluated for surgical intervention immediately.

## Introduction

There are 10-15,000 new cases of infective endocarditis (IE) in the United States every year. Patients that are at high risk of endocarditis include intravenous drug users, congenital heart disease or prosthetic valves, poor dentition, and end-stage renal disease on hemodialysis [1]. The most common pathogens that cause endocarditis are the Staphylococcus species. Due to poor prognosis of patients with endocarditis, early diagnosis and management of these patients can be challenging in the clinical setting. Positive blood cultures can take up to a few days to result, and unless there is a significant new murmur, endocarditis is a diagnosis that is looked at after other sources of infection are ruled out. In-hospital mortality can be up to 22% with a five-year mortality rate of 45% in cases that predominantly encompass endocarditis in one or possibly two heart valves [2].

Most cases of IE usually affect one or two valves of the heart with the most common being the tricuspid valve. The tricuspid valve is the first valve contacted when venous blood supply returns to the heart and IV drug use is a frequent cause of bacteremia [3,4]. Very rarely we do see cases that affect all three valves of the heart. We present a case of a patient with triple valve endocarditis complicated by abscess formation, perforation, and complete heart block secondary to a diabetic foot ulcer.

## Case presentation

**Figure 1:**
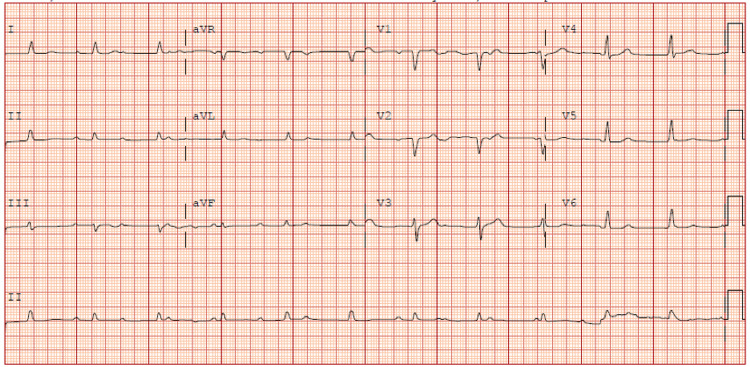
Electrocardiogram showing complete heart block

A 45-year-old man with a past medical history of type 2 diabetes mellitus with a non-healing right foot ulcer was found at home collapsed on the floor with altered mental status and weakness. His Glasgow Coma Scale (GCS) score was 10. The patient was normocardic and normotensive on presentation but was tachypneic and had a fever of 103.1 °F. The patient was intubated upon emergency department admission for airway protection. Initial electrocardiogram (EKG) revealed normal sinus rhythm with prolonged QT interval of 518 ms. STAT computed tomography (CT) of the brain was unremarkable for any acute intracranial process. The patient’s initial labs were significant for leukocytosis of 20,000 cells/ml^3^, lactate of 2.0 mmol/L, procalcitonin of 6.43 ng/mL, troponin of 0.22 ng/ml, and labs supportive of diabetic ketoacidosis. Troponins were trended and peaked at 0.41 ng/ml. A subsequent MRI of the brain was significant for multiple, widespread abnormal foci highly suggestive of an underlying embolic phenomenon with acute/subacute ischemic infarctions. A transthoracic echocardiogram (TTE) was performed and revealed left ventricular dysfunction with an ejection fraction of 45-50% and vegetations on the aortic and mitral valve. A subsequent EKG showed complete third-degree heart block which rose suspicion for possible valvular abscess formation (Figure 1).

**Figure 2:**
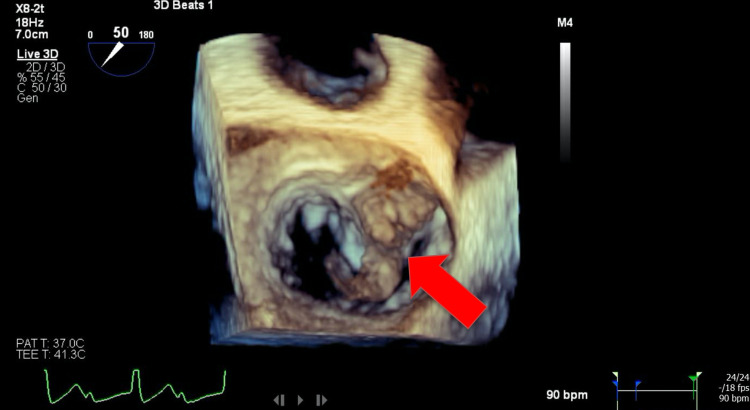
Transesophageal echocardiogram 3D image showing anterior mitral leaflet vegetation (red arrow)

**Figure 3:**
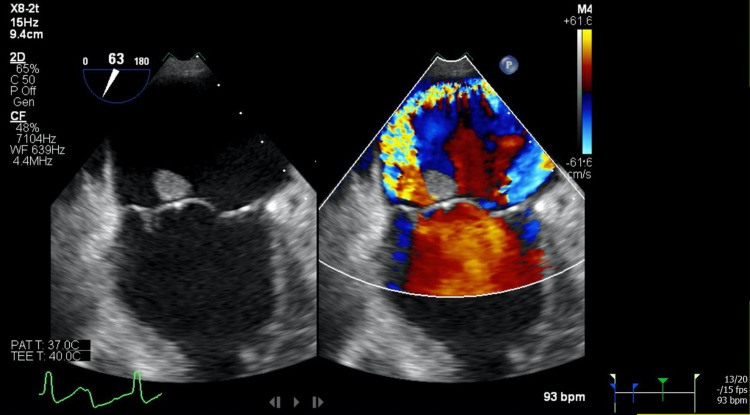
(Left) Transesophageal echocardiogram showing large mitral valve vegetation; (right) transesophageal echocardiogram with color doppler showing severe mitral regurgitation through the valve and perforated anterior leaflet

**Figure 4:**
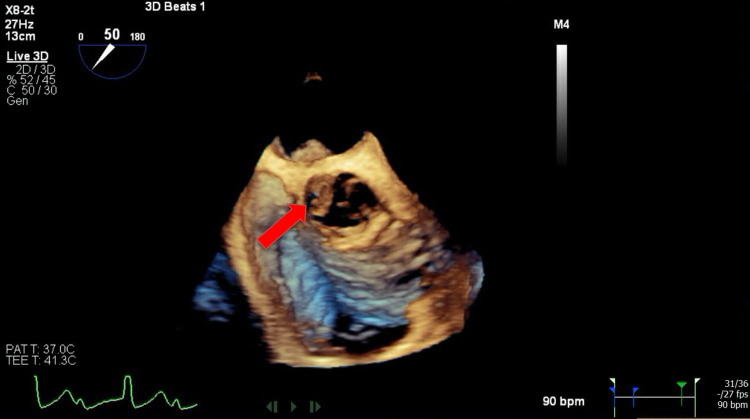
Three-dimensional aortic valve image showing big vegetation (red arrow)

**Figure 5:**
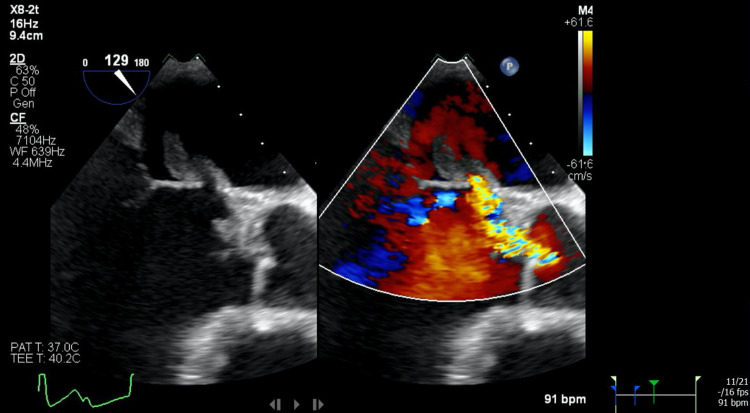
(Left) Transesophageal echocardiogram image showing large vegetation on the aortic and mitral valve; (right) transesophageal echocardiogram with color doppler showing severe aortic regurgitation

**Figure 6:**
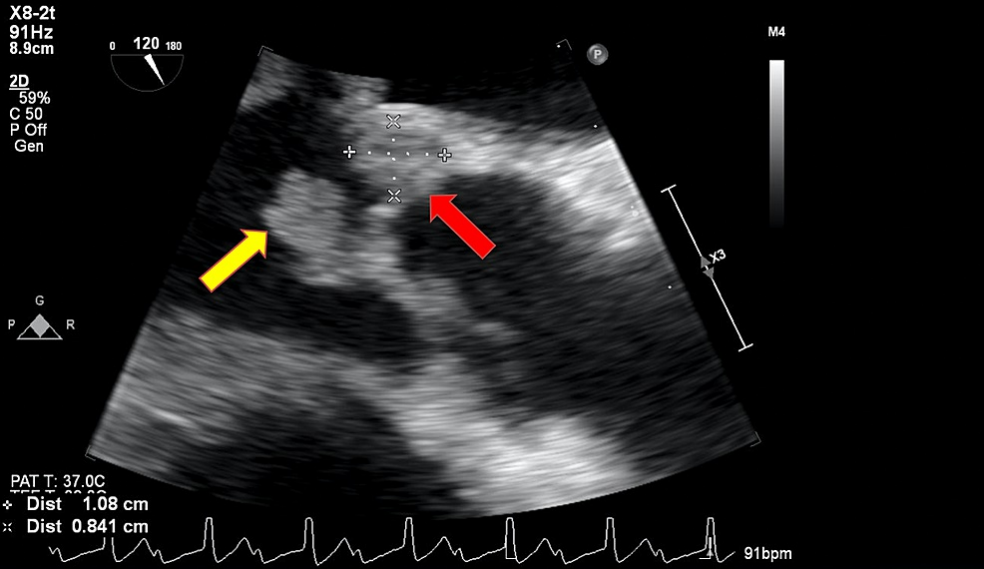
Transesophageal echocardiogram showing a large aortic root abscess (red arrow) and aortic valve vegetation (yellow arrow)

**Figure 7:**
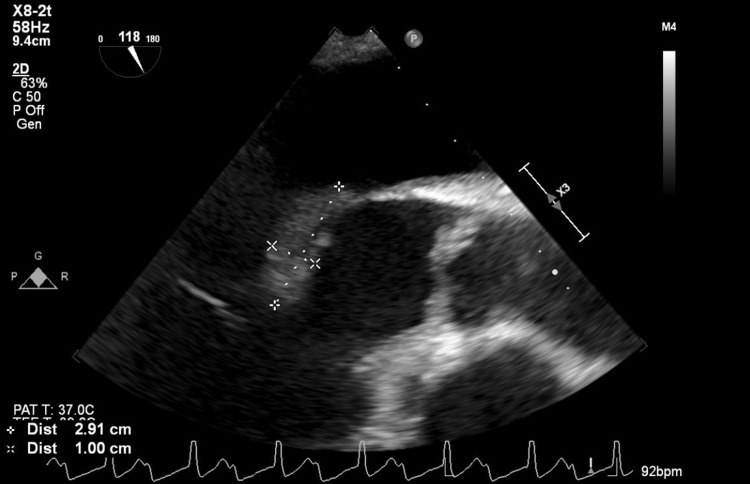
Transesophageal echocardiogram image showing large mitral valve vegetation

A transesophageal echocardiogram (TEE) with 3D reconstruction revealed severe aortic regurgitation, multiple vegetations (up to 1.6 × 0.8 cm^2^) present on the bicuspid aortic valve, and a moderately sized aortic valve abscess likely contributing to the patient’s heart block. It also revealed severe mitral regurgitation, a large and mobile anterior mitral leaflet vegetation (1.9 × 0.8 cm^2^), along with perforation of the anterior mitral leaflet. The tricuspid valve also had a small vegetation and mild regurgitation that was not previously seen on TTE (Figures 2-7).

The patient did not have a history of IV drug abuse or rheumatic heart disease. Blood cultures were also positive for *Streptococcus agalactiae* (GBS) and *Escherichia coli* which supported the patient’s diagnosis of triple valve endocarditis likely secondary to an infected foot ulcer. The patient was then immediately transferred to a quaternary care center where he eventually underwent surgery after ruling out mycotic aneurysm. On surgical exploration, he was noted to have perforation of the right coronary cusp and anterior mitral valve leaflet with vegetations and abscess formation in the central fibrous body between the valves. He underwent aortic valve, mitral valve, and aortic root debridement with aortic valve replacement and mitral valve repair with annuloplasty band and autologous pericardial patch. The patient tolerated the surgery well. He was unable to be weaned from the ventilator and ultimately underwent placement of a tracheostomy tube. He was later discharged to a long-term acute care center for further recovery with a prolonged antibiotic course with ceftriaxone.

## Discussion

Infective endocarditis is characterized by inflammation and infection of the endocardium of the heart and typically involves one or more heart valves or an intracardiac device. While many patients with IE have risk factors such as rheumatic heart disease, IV drug use, structural or congenital abnormalities, or prosthetic valves, this patient did not have any known risk factors [1]. The only possible source of infection in this case was the patient’s diabetic foot ulcer. Additionally, a majority of cases of IE only involve one of the heart valves. Involvement of two valves is uncommon, and three is even rarer. Among known cases of multi-valve IE, left-sided lesions are more common with very few showing involvements in both left- and right-sided lesions. The higher incidence of left-sided lesions can be attributed to higher turbulent flow across valves and higher prevalence of acquired and congenital anomalies of left-sided valves. On the other hand, IV drug use is associated with a majority of right-sided lesions [5].

Diagnosis of endocarditis was made using the currently accepted criteria, the modified Duke criteria [6]. In this case, both major criteria were met - positive blood cultures and evidence of endocardial involvement on echocardiogram. Once the diagnosis was made, it was important to determine the number of valves involved. Studies show that multi-valve endocarditis has an increased risk of complications such as congestive heart failure, acute renal failure, embolic events (pulmonary embolism, cerebral vascular accident, disseminated intravascular coagulation, etc.), and increase in splenic abscess/infarct [7]. Our patient developed native triple valve endocarditis from GBS and *E. coli* bacteremia and echocardiographic evidence of aortic, mitral, and tricuspid valve involvement complicated by aortic root abscess, complete heart block, acute hypoxic respiratory failure, and multiple septic emboli.

Due to the high prevalence of serious complications of multi-valve IE and increased risk of in-hospital mortality due to persistent infection, these patients should be considered for urgent surgical treatment. The American College of Cardiology/American Heart Association (ACC/AHA) have certain indications for surgical treatment of native valve IE, including moderate to severe heart failure, severe aortic or mitral regurgitation with hemodynamic instability, perivalvular infection with abscess formation, and embolic events despite antibiotic therapy. On the other hand, surgical treatment for right-sided IE is generally avoided and only considered for surgery if the organism is difficult to eradicate or there is a tricuspid valve vegetation greater than 20 mm that persists after recurrent pulmonary emboli [8]. Annular abscess formation is one of the major factors that makes multi-valve surgery technically more complex, affecting early mortality. Specially, when fibrous structure between the aortic and mitral valve - intervalvular fibrosa (IVF) is involved with abscess formation, radical surgery comprising debridement, valve replacement, and reconstruction of IVF is indicated. These surgeries have been referred as “Commando Procedure,” “Hemi-Commando Procedure,” or “UFO Procedure” by different groups [9]. Factors associated with late mortality include the presence of prosthetic and early endocarditis, aortic valve involvement, emboli, congestive heart failure, and Staphylococcal infection and renal failure [10].

Given the extent of endocarditis involving all three valves, aortic root abscess, complete heart block and septic emboli, this patient had multiple indications for surgical intervention. Although there is no consensus for optimal timing of early surgery, the rapid decision to transfer to a quaternary care center for urgent surgical intervention was indicated in this patient. Imaging played a distinguished role in detecting early and late mechanical complications of endocarditis, assessing cardiac function, and guiding emergent clinical decision-making.

## Conclusions

Infective endocarditis has a poor prognosis and high mortality rates, consequently the prognosis is poorer in patients with multiple valve involvement. The prompt utilization of TEE in detecting early and late mechanical complications of endocarditis is imperative in facilitating rapid clinical decision-making and early intervention. Patients with multi-valve endocarditis are at extremely high risk of complications and the patient should be evaluated for surgical intervention immediately.
